# Neural circuits mediating olfactory-driven behavior in fish

**DOI:** 10.3389/fncir.2013.00062

**Published:** 2013-04-11

**Authors:** Florence Kermen, Luis M. Franco, Cameron Wyatt, Emre Yaksi

**Affiliations:** ^1^Neuroelectronics Research FlandersLeuven, Belgium; ^2^Vlaams Instituut voor BiotechnologieLeuven, Belgium; ^3^KU Leuven, LeuvenBelgium

**Keywords:** teleost, zebrafish, anatomy and physiology, behavior, olfactory bulb, olfactory epithelium, habenula, hypothalamus

## Abstract

The fish olfactory system processes odor signals and mediates behaviors that are crucial for survival such as foraging, courtship, and alarm response. Although the upstream olfactory brain areas (olfactory epithelium and olfactory bulb) are well-studied, less is known about their target brain areas and the role they play in generating odor-driven behaviors. Here we review a broad range of literature on the anatomy, physiology, and behavioral output of the olfactory system and its target areas in a wide range of teleost fish. Additionally, we discuss how applying recent technological advancements to the zebrafish (*Danio rerio*) could help in understanding the function of these target areas. We hope to provide a framework for elucidating the neural circuit computations underlying the odor-driven behaviors in this small, transparent, and genetically amenable vertebrate.

## INTRODUCTION

Teleosts, the infraclass to which zebrafish belong, account for nearly half of all extant vertebrate species. The diversity of forms in these closely related species provide opportunities to study similar but distinct brain organizations and behavioral programs. Due to this, there already exists a wealth of literature on the teleost olfactory system, pre-dating many genetic and optical techniques, in such members as goldfish and catfish. Despite these variations, the architecture of the zebrafish olfactory system is fundamentally similar to that of other vertebrates. On the molecular level, families of receptor proteins expressed by the olfactory sensory neurons are comparable within most vertebrates, with zebrafish possessing a modest repertoire several times smaller than that of mammals (Alioto and Ngai, 2005).

The olfactory system is of particular relevance to systems neuroscience due to the large variety of stimuli that need to be encoded as well as the simple but interesting computations it performs, such as gain control, pattern decorrelation, categorization, and detecting weak stimuli despite highly dynamic background “noise.” Furthermore, olfactory stimuli can trigger a wide range of behaviors related to reproduction, appetite, fear, and anxiety, which allow the study of the brain circuits that are involved in generating these essential behaviors. Finally, the activity patterns evoked by these odors can be readily recorded in highly conserved structures of the olfactory system, i.e., the olfactory epithelium, the olfactory bulb, and olfactory telencephalic and diencephalic centers, owing to the accessibility of these brain regions in zebrafish.

Over the past decade, the zebrafish (*Danio rerio*) has become increasingly popular in systems neuroscience. The success of this model organism is mainly due to its small brain that is amenable to functional imaging and genetic manipulations. The extensive genetic toolbox of the zebrafish can readily be combined with optical and electrophysiological techniques and quantitative behavioral assays to perform experiments that were impossible only a few years ago. Here we review a wide range of literature on the anatomy and the function of the olfactory system in zebrafish and other teleosts and discuss how the novel experimental tools of the zebrafish can and will transform this field. We hope to provide a framework for elucidating the neural circuit computations underlying the odor-driven behaviors in this small, transparent, and genetically amenable vertebrate.

## ODORANTS SENSED BY FISH

The fish olfactory system can detect a wide range of water soluble compounds which elicit, or contribute to, behaviors crucial for survival such as feeding, reproduction, social interaction, and avoiding predation. Amino acids and nucleotides indicate the presence of food. Nucleotides, such as adenosine-5′-triphosphate (ATP), indicate food freshness in carp (Hara and Zielinski, 2007). Amino acids induce appetitive swimming behavior characterized by increased number of turns and swimming speed in zebrafish (Lindsay and Vogt, 2004). Steroids and prostaglandin F2α, which are hormones produced in the gonads and released in urine, were shown to trigger species and sex specific reproductive behaviors in a variety of teleosts (Hurk and Lambert, 1983; Stacey and Kyle, 1983). Bile acids are steroids secreted by the liver and released in urine, which have been implicated in migration to spawning sites in lampreys (Sorensen et al., 2005). While bile acids are agreed upon as one of the main classes of odorant in fish, their putative role as social pheromones, indicating the presence of other fish, is not yet conclusively proven in teleosts (Doving et al., 1980). Compounds released from the skin of injured fish have long been known to elicit a vigorous, stereotyped alarm response from many species of fish (von Frisch, 1941). This alarm response is characterized by darting followed by slow swimming or freezing (Speedie and Gerlai, 2008; Doving and Lastein, 2009).

## OLFACTORY EPITHELIUM

Odorants are detected upon interaction with olfactory receptors (ORs) in the nose. Teleosts have two nasal cavities, one on each side of the head at the extremity of the snout (Hansen and Zielinski, 2005). Unlike in mammals, there is no sniffing in teleost fish. Each nasal cavity is composed of an anterior nostril, through which water enters the nose, and a posterior nostril, through which water exits the nose. The olfactory epithelium lies between these two nostrils (Hara and Zielinski, 2007). In zebrafish, it is multilamellar and rosette-shaped. Zebrafish olfactory sensory neurons are comprised of three morphologically distinct types of cells: (1) ciliated cells, with long dendrites and few cilia, (2) microvillous sensory neurons, with shorter dendrites and microvilli, (3) crypt cells, pear-shaped cells specific to fish, with microvilli and few cilia (Hansen and Zielinski, 2005). While ciliated and microvillous cells are present in higher vertebrates, crypt cells have only been found in fish (Hansen et al., 1999; Schmachtenberg, 2006; Vielma et al., 2008). The soma of olfactory sensory neurons are located at different depths in the olfactory epithelium: ciliated cells are situated in the deep layer, microvillous cells are located in the intermediate layer and mature crypt cells are located in the most superficial layer, forming the pseudo-stratified structure of the olfactory epithelium. Scattered amongst the olfactory sensory neurons are ciliated non-sensory cells, which help to move the mucus covering the olfactory epithelium (Zeiske et al., 1992). Crypt, microvillous, and ciliated cells are dispersed throughout the epithelium. They represent respectively 2, 8, and 90% of the total olfactory sensory neuron population, in trout and mackerel (Sato and Suzuki, 2001; Schmachtenberg, 2006). Olfactory sensory neurons are constantly renewed throughout adult life or following chemical lesion of the epithelium (Cancalon, 1982; Julliard et al., 1996; Bettini et al., 2006). This regeneration is mediated by the division of basal cells located in the deepest layer of the olfactory epithelium (Cancalon, 1982).

In fish, as in mammals, the detection of odorants by olfactory sensory neurons is mediated by different families of G-protein-coupled receptors. The zebrafish genome contains 143 OR genes, 56 vomeronasal receptor (VR) genes, and 109 trace amine-associated receptor (TAAR) genes (Alioto and Ngai, 2005; Hashiguchi and Nishida, 2006, 2007; Saraiva and Korsching, 2007). Ciliated cells express ORs whereas microvillous cells express VRs (Yoshihara, 2009). The precise identity of the receptor mediating the odor response in crypt cells is not known. However, a recent study found that crypt cells express a member of the VR family in zebrafish (Oka et al., 2012). Subsets of zebrafish olfactory sensory neurons express members of the TAAR gene family (Hussain et al., 2009).

As in other vertebrates, most olfactory sensory neurons express only one receptor (Sato et al., 2007). As a consequence, the response profile of a given neuron is constrained by the receptive field of the receptor it expresses. Patch clamp recordings of neurons isolated from fish olfactory epithelium provided insights into the repertoire of ligands that bind to ORs and VRs. In channel catfish, both ciliated and microvillous cells respond to amino acids (Sato and Suzuki, 2001; Hansen et al., 2003; Schmachtenberg and Bacigalupo, 2004). Ciliated cells also respond to urine extracts containing bile acids and might be involved in alarm substance detection (Sato and Suzuki, 2001; Doving and Lastein, 2009). Nucleotides activate microvillous cells (Hansen et al., 2003). However, the ligands of crypt cells have proven more elusive. Since their discovery, crypt cells have been hypothesized to participate in reproductive pheromone detection. Their density and depth in the olfactory epithelium was shown to vary depending on the seasons in sexually mature carp (Hamdani el et al., 2008). Moreover crypt cell density is sex-dependent in certain fish species (Bettini et al., 2012). A large majority of crypt cells respond to only one category of odorants. Intracellular recordings and calcium imaging studies carried out on mackerel and juvenile trout showed that different subsets of crypt cells respond either to amino acids, bile acids, or reproductive pheromones (Schmachtenberg, 2006; Vielma et al., 2008; Bazaes and Schmachtenberg, 2012). However, in mature trout, the majority of crypt cells respond to reproductive pheromones of the opposite sex and not to the other categories, indicating a change in the response profile of crypt cells during life, depending on sexual maturity and sex of the fish (Bazaes and Schmachtenberg, 2012).

**FIGURE 1:**
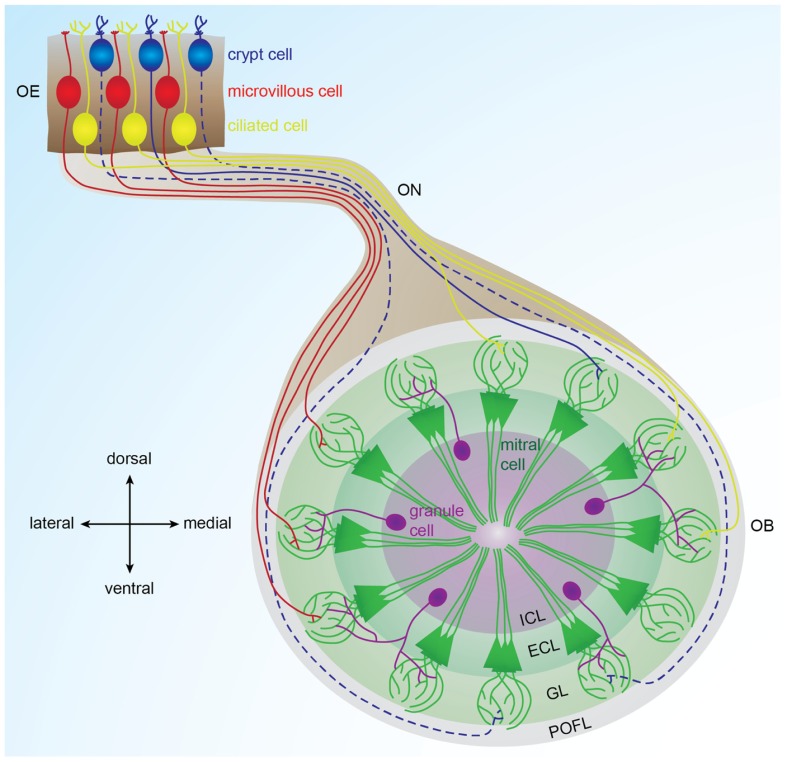
**Organization of the olfactory bulb network**. Odorants are detected in the olfactory epithelium by three types of sensory neurons (microvillous, ciliated, and crypt cells) that project to different glomeruli located in different areas of the olfactory bulb. Ciliated cells mainly project to the dorsal and medial olfactory bulb; microvillous cells project to the lateral olfactory bulb (Sato et al., 2005, 2007). Crypt cells project to a dorsomedial domain in zebrafish and to a ventral domain in carp (Hamdani el and Doving, 2006; Gayoso et al., 2012). In each glomerulus, olfactory sensory neuron axons contact dendrites of mitral cells, the output cells of the olfactory bulb. Inhibitory interneurons called granules cells are located in the deepest layer of the bulb and modulate the activity of mitral cells. Solid lines represent connections described in zebrafish. Dotted lines represent connections described in other fish species. OE: olfactory epithelium, OB: olfactory bulb, ON: olfactory nerve, POFL: primary olfactory fiber layer, GL: glomerular layer, ECL: external cell layer, ICL: internal cell layer.

As in other vertebrates, zebrafish olfactory sensory neurons expressing the same receptor are dispersed throughout the epithelium (Baier et al., 1994; Weth et al., 1996). They project their axons via the olfactory nerve to the same glomerulus in the ipsilateral olfactory bulb (Hansen and Zielinski, 2005; Sato et al., 2007). Moreover, the bulbar projection pattern of the three types of olfactory sensory neurons shows a coarse spatial organization. Using a double transgenic zebrafish line labeling ciliated and microvillous cells with different fluorophores, studies have shown that ciliated cells mainly project to the dorsal and medial olfactory bulb, whereas microvillous cells project to the lateral olfactory bulb (Sato et al., 2005, 2007). Retrograde labeling of the olfactory epithelium following lipophilic tracer application to different bulbar domains showed that crypt cells project to the ventral olfactory bulb in carp and to the dorsomedial olfactory bulb in zebrafish (Hamdani el and Doving, 2006; Gayoso et al., 2012). This projection pattern, shown in **Figure 1**, is well-conserved between the two bulbs of the same zebrafish, as well as among individual zebrafish (Baier and Korsching, 1994; Braubach et al., 2012).

Additionally, a subset of fibers originating from the nose reach the telencephalon without contacting the olfactory bulb in several teleosts (Honkanen and Ekstrom, 1990; Riddle and Oakley, 1992; Gayoso et al., 2011). Extrabulbar primary olfactory projections to telencephalic centers have also been described in amphibians but not in mammals (Pinelli et al., 2004). In white sturgeon, these fibers terminate in the posterior tubercle, a diencephalic region (Northcutt, 2011). In trout, these fibers innervate the ventral nucleus of the ventral telencephalon (Vv) and the dorsal telencephalon, as well as the preoptic area and the hypothalamus (Becerra et al., 1994; Anadon et al., 1995). In zebrafish, lipophilic tracer application in the Vv retrogradely labels a few bipolar olfactory sensory neurons in the olfactory epithelium, indicating that ciliated and/or microvillous cells send direct projections to Vv (Gayoso et al., 2011). Nevertheless, the functional role of these extrabulbar primary connections remains unknown.

## THE OLFACTORY BULB: PRIMARY PROCESSING OF ODOR INFORMATION

The olfactory bulb is the vertebrate brain structure that receives the large majority of olfactory sensory neuron inputs through the olfactory nerve. Understanding the neurophysiological mechanisms governing odor processing in the olfactory bulb requires a profound comprehension of its neuronal connectivity and physiological properties. In zebrafish, the olfactory bulb is comprised of approximately 20,000 neurons (Friedrich et al., 2009) organized in four concentric layers (**Figure 1**). From superficial to deep, these are: (1) primary olfactory fiber layer, formed by olfactory sensory neuron axons (Sato et al., 2007); (2) glomerular layer, containing approximately ≈140 spherical modules of neuropil named glomeruli (Braubach et al., 2012); (3) external cell layer, consisting of mitral and ruffed cell somas (Fuller and Byrd, 2005; Fuller et al., 2006); and (4) internal cell layer, containing cell bodies of different interneurons, namely juxtaglomerular, periglomerular, and granular cells (Edwards and Michel, 2002; Bundschuh et al., 2012).

Glutamatergic mitral and ruffed cells are the principal cells of the olfactory bulb in fish (Edwards and Michel, 2002). In zebrafish, apical dendrites of mitral cells receive direct synaptic inputs from olfactory sensory neurons in glomeruli and project to the telencephalon and diencephalon (Fuller et al., 2006; Miyasaka et al., 2009). Teleost ruffed cells are not innervated by olfactory sensory neurons. Nevertheless, ruffed cells receive synaptic contacts from mitral cells and bulbar interneurons (Kosaka and Hama, 1979, 1981, 1982; Kosaka, 1980).

Interneurons are localized deeper in the olfactory bulb. They mediate lateral interactions within bulbar neurons. The ratio of interneurons to mitral cells is 10:1 in zebrafish (Wiechert et al., 2010), whereas in mammals it is 100:1 (Rosselli-Austin and Altman, 1979). In zebrafish, GABAergic granule cells, which lack axons, are located in the inner layer of the olfactory bulb and extend their processes to make dendrodendritic synaptic connections with principal cells. Juxtaglomerular and periglomerular cells are apposed to glomeruli and express glutamate and dopamine, respectively, in addition to GABA (Byrd and Brunjes, 1995; Edwards and Michel, 2002).

### ODOR CODING IN THE OLFACTORY BULB

Each glomerulus receives convergent input from olfactory sensory neurons expressing the same odorant receptor (Sato et al., 2005, 2007). Individual odorant receptors respond to different odors and a given odor generally activates several odorant receptors. As a consequence, odor stimulation in zebrafish and goldfish activates spatially distributed ensembles of glomeruli (Friedrich and Korsching, 1997, 1998; Speca et al., 1999; Fuss and Korsching, 2001). Glomeruli responding to similar molecular features are organized into defined zones within the olfactory bulb, forming chemotopic maps. Yet, odorants frequently activate glomeruli beyond their chemotopical domain. As a consequence, odorants are represented as fractured maps in the olfactory bulb (Friedrich and Korsching, 1997, 1998). In zebrafish, first-order chemical features, such as molecular categories, are encoded by large glomerular domains. Second-order features, such as carbon chain length or branching, are encoded by local differences of glomerular activity patterns within chemotopical domains (Friedrich and Korsching, 1997, 1998; Fuss and Korsching, 2001; Korsching, 2001). Chemotopic maps are therefore hierarchically organized such that fine maps of secondary features are nested within coarse maps of primary features (Friedrich and Korsching, 1997, 1998).

In zebrafish, the lateral subregion of the olfactory bulb responds preferentially to amino acids and to nucleotides, whereas the medial subregion responds to bile acids (Friedrich and Korsching, 1997, 1998; Koide et al., 2009). Genetic ablation of subsets of synaptic inputs to the olfactory bulb from the olfactory epithelium has revealed that the lateral glomerular cluster is responsible for feeding behavior evoked by amino acids (Koide et al., 2009). Fish skin extract is a mixture of several compounds that trigger alarm responses in zebrafish and one of its components is shown to activate mediodorsal posterior and anterolateral olfactory glomeruli (Mathuru et al., 2012). In addition, a group of ventral glomeruli responds to prostaglandin (Friedrich and Korsching, 1998). Amino acids, bile acids, and nucleotides evoke combinatorial glomerular activity patterns that overlap but are sufficiently complex so that even very similar odorants can be discriminated. In contrast, pheromones are represented in a non-combinatorial fashion, suggesting a direct relay to brain structures controlling sex behavior and endocrine states (Friedrich and Korsching, 1997, 1998).

In goldfish, ruffed cells are spontaneously active, and are inhibited by granule cells which are activated by mitral cells (Zippel et al., 1999). Upon odor stimulation, mitral cells respond with high frequency burst-like firing rates triggered by olfactory sensory neuron activity, whereas ruffed cell firing rates are low. Moreover, mitral and ruffed cells frequently respond with contrasting activity patterns. Excitation of mitral cells drives ruffed cell inactivation via granule cells, and inhibition of mitral cells releases ruffed cells from inactivation (Zippel et al., 1999).

In zebrafish, mitral cell activity patterns are dynamically reorganized during the initial phase (~400 ms) of an odor response before they reach a steady state (Friedrich and Laurent, 2001, 2004). Mitral cell activity patterns evoked by similar odorants overlap during the early phase of the odor response but subsequently diverge. Hence initially overlapping odor responses decorrelate over time (Friedrich and Laurent, 2001, 2004). It has been suggested that mitral cell firing patterns convey multiplexed information about odors simultaneously (Friedrich et al., 2004). This study proposed that the mitral cell action potentials, which are phase-locked to the local field potential oscillations, carry information about the odor category and the remaining mitral cell activity informs about precise odorant identity. Thus, multiplexed mitral cell activity patterns simultaneously convey information about complementary odorant features (Friedrich et al., 2004). Although glomerular responses to different odors are highly variable, total mitral cell firing remains within a relatively narrow range, suggesting a gain control, probably through inhibitory circuits (Friedrich and Laurent, 2004; Friedrich et al., 2009).

### SYNAPTIC INPUTS TO THE OLFACTORY BULB FROM HIGHER BRAIN AREAS

In zebrafish, the olfactory bulb receives serotonergic inputs from raphe nuclei (Lillesaar et al., 2009) and cholinergic inputs through the terminal nerve ganglion (Edwards et al., 2007). In rodents, serotonin and acetylcholine increase the activity of interneurons while reducing the excitability of principal cells (Castillo et al., 1999; Ghatpande et al., 2006; Pressler et al., 2007; Petzold et al., 2009; Liu et al., 2012). In carp, noradrenaline enhances postsynaptic long term potentiation evoked by tetanic stimulation of mitral cell–granule cell synapses (Satou et al., 2006). In addition, centrifugal fibers originating from the telencephalon terminate in the olfactory bulb internal cell layer of teleosts, probably making synaptic contact with granule cells, raising the possibility that cortical feedback modulates the bulbar network (Munz et al., 1982; Stell et al., 1984; Zucker and Dowling, 1987). Nevertheless, further studies are needed in order to elucidate the physiological role of these neuromodulators onto bulbar neural circuits in fish.

### ORGANIZATION OF OLFACTORY BULB PROJECTIONS

**FIGURE 2:**
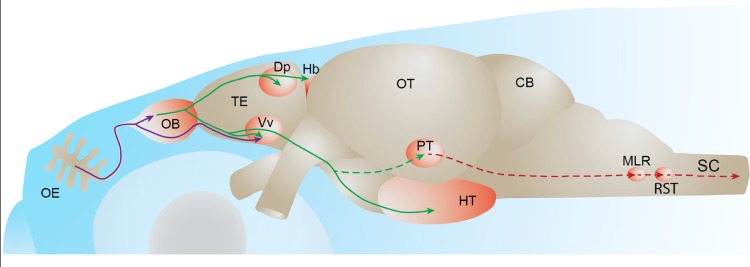
**Fish olfactory system**. Primary projections from olfactory sensory neurons to the olfactory bulb or telencephalon are depicted in purple. Secondary olfactory projections from the olfactory bulb to the telencephalon and diencephalon are depicted in green. A putative olfacto-motor pathway connecting the posterior tubercle to executive motor centers in the mesencephalon, described in lampreys, is depicted in red. Solid lines represent connections described in zebrafish. Dotted lines represent connections described in other fish species. OE: olfactory epithelium, OB: olfactory bulb, TE: telencephalon, Dp: dorsal-posterior part of the telencephalon, Hb: habenula, Vv: ventral nucleus of the ventral telencephalon, OT: optic tectum, PT: posterior tubercle, HT: hypothalamus, CB: cerebellum, MLR: mesencephalic locomotor region; RST: reticulo-spinal tract, SC: spinal cord.

Mitral cells extend their axons through the medial and lateral olfactory tracts to different higher brain centers (**Figure 2**). In carp and zebrafish, the lateral olfactory tract contains mainly fibers originating in the lateral olfactory bulb, whereas the medial olfactory tract contains mainly fibers originating from the medial olfactory bulb (Sheldon, 1912). Anatomical tracing studies have shown that the teleost medial olfactory tract is subdivided into medial and lateral regions (Sheldon, 1912; Finger, 1975; Bass, 1981; von Bartheld et al., 1984). The lateral part of the medial olfactory tract is comprised largely of centrifugal fibers projecting to the olfactory bulb whereas the medial part of the medial olfactory tract as well as the lateral olfactory tract contain mitral cell axons projecting to telencephalic and diencephalic areas (von Bartheld et al., 1984). In addition, the medial part of the medial olfactory tract carries mitral cell axons that project to the contralateral olfactory bulb (von Bartheld et al., 1984).

The medial and lateral olfactory tracts are separate, anatomically well-defined axon bundles, which enables the study of their physiological function by several experimental manipulations across different fish species (Stacey and Kyle, 1983; Hamdani et al., 2000, 2001). It was shown that the electrical stimulation of the medial olfactory tract induces alarm reaction or reproductive behavior, while lateral olfactory tract stimulation induces feeding behaviors in cod (Doving and Selset, 1980). These different functions could arise from different projection profiles of these two tracts to higher brain centers. In the goldfish, fibers carried by the medial and lateral olfactory tracts reach target areas in the telencephalon and the posterior tubercle of the diencephalon (von Bartheld et al., 1984). This study also showed that the lateral olfactory tract specifically innervates the habenula while the medial olfactory tract also sends projections to the Vv. These projection patterns have been confirmed by anatomical tracing studies in other fish species (Huesa et al., 2000; Folgueira et al., 2004; Northcutt, 2011). In zebrafish, the mitral cells are shown to project to Vv and the dorsal-posterior part of the telencephalon (Dp) and to the right habenula and the hypothalamus in the diencephalon (Rink and Wullimann, 2004; Miyasaka et al., 2009; Gayoso et al., 2011).

## OLFACTORY BULB TARGETS

### TELENCEPHALON

Dp in teleosts corresponds to the mammalian primary olfactory (piriform) cortex, whereas teleost Vv is the homolog of the septal area, a part of the limbic system, in mammals. Whether the chemotopic odor maps in the olfactory bulb are maintained in Dp and Vv in fishes remains a subject of debate. Recording of single neurons in the channel catfish pallium showed a spatial segregation of neurons preferentially responding to odorants belonging to the same biological categories (Nikonov et al., 2005). This study showed that bile acids preferentially activate the medial pallium whereas amino acids and nucleotides preferentially activate the lateral pallium (comprising Dp), indicating a gross chemotopical organization in the telencephalic targets of the olfactory bulb. However, a recent functional imaging study suggested that the spatial segregation of odor responses was not prominent in Vv and Dp neurons of zebrafish (Yaksi et al., 2009). This study showed that Vv and Dp display overlapping and distributed activity in response to various odor categories (bile acids, amino acids, nucleotides). Hence odor representations in the telencephalon do not display strong chemotopy (although slight differences between the distribution of amino acid and bile acid-evoked activity can be observed in the Dp). This is in accordance with work in rodents, where optical imaging in the mouse primary olfactory cortex shows that odor-evoked activity is not spatially segregated in the main bulbar target (Stettler and Axel, 2009).

Vv and Dp neurons were shown to have different response properties. Vv neurons are broadly tuned resulting in overlapping representation of odor categories, whereas Dp neurons respond to odors more specifically (Yaksi et al., 2009). The activity of mitral cell ensembles was shown to carry multiplexed information about stimulus features such as category and identity (Friedrich et al., 2004). How is the multiplexed output provided by the olfactory bulb decrypted in the telencephalic targets? Dp cells were shown to be relatively insensitive to oscillatory mitral cell activity, which informs about odor categories (Blumhagen et al., 2011). This study suggests that Dp establishes specific and decorrelated odor representations. However a previous study suggests that the pattern correlation in Dp neurons is not significantly different from the pattern correlation in mitral cells (Yaksi et al., 2009). Further studies are needed to examine whether the multiplexed olfactory bulb output is read and used by its targets.

Importantly, Vv and Dp receive substantial neuromodulatory inputs which could participate in odor response refinement in these areas. In zebrafish, the pallium (comprising Dp) and the subpallium (comprising Vv) share inputs from locus coeruleus (noradrenergic), raphe nuclei (serotoninergic), and posterior tubercle (dopaminergic; Rink and Wullimann, 2004; Scharer et al., 2012). The subpallium additionally receives inputs from the cholinergic superior reticular nucleus and the histaminergic caudal hypothalamus (Rink and Wullimann, 2004). It was shown that dopamine selectively decreases inhibitory but not excitatory odor responses in the Dp (Scharer et al., 2012). Calcium imaging further showed that the amplitude of odor responses was increased in the presence of dopamine, without affecting the spatial response pattern. Therefore, dopamine mediated increase of odor response gain might mediate changes in odor saliency during learning.

### DIENCEPHALON

#### Habenula

The habenula is a highly conserved brain region that connects the forebrain to brainstem nuclei such as the interpeduncular nucleus, the serotonergic raphe nuclei and the ventral tegmental area containing dopaminergic neurons (Tomizawa et al., 2001; Hikosaka, 2010). The habenula is divided into two parts based on connectivity and functional heterogeneity: the medial and lateral mammalian habenulae, which are homologous to the dorsal and ventral fish habenulae, respectively (Amo et al., 2010). It was shown in several teleost species that mitral cells project to the habenula (Wedgwood, 1974; von Bartheld et al., 1984; Miyasaka et al., 2009; Northcutt, 2011). In zebrafish, bulbar projections to the habenula are asymmetric. Indeed, it has been shown that mitral cells located in both olfactory bulbs send axons that terminate in the medial compartment of the right habenula (Miyasaka et al., 2009).

The mammalian homolog of the fish ventral habenula has been proposed to participate in the control of motor behaviors depending on stimulus values by influencing the activity of dopaminergic neurons (Hikosaka, 2010). Moreover, two recent studies showed that when the dorsal habenula is genetically inactivated, zebrafish display altered responses to conditioned fear stimuli (Agetsuma et al., 2010; Lee et al., 2010). These studies indicate a role for the habenula in experience-dependent modulation of fear responses. The role of habenula in odor processing and the functional architecture of its circuitry remain to be uncovered.

#### Posterior tubercle

The posterior tubercle is a ventral region of the posterior diencephalon. Because the posterior tubercle contains dopaminergic cells, it has been proposed to be functionally similar to the mammalian mesolimbic dopaminergic system (Rink and Wullimann, 2001). Bulbar efferents have been shown to terminate in the posterior tubercle of several teleost fish (Matz, 1995; Von Bartheld, 2004; Derjean et al., 2010; Northcutt, 2011; Northcutt and Rink, 2012). However, it is currently not known whether this projection also exists in zebrafish.

A recent work suggested that the projections from the olfactory bulb to the posterior tubercle play a role in the generation of olfactory-driven locomotor activity in the sea lamprey (Derjean et al., 2010). This study showed that stimulation of the medial olfactory bulb by glutamate injection generated rhythmical electrical activity in reticulospinal cells and in the ventral root of the spinal cord, which resembles fictive locomotion. The proposed olfacto-motor pathway is comprised of a medial glomerulus projecting to the posterior tubercle, which would then transmit the olfactory input to the mesencephalic locomotor region that in turn excites reticulospinal cells which are command neurons responsible for the activation of spinal locomotor networks. This study is the first demonstration of a functional connection between the olfactory system and the spinal locomotor network in vertebrates.

#### Hypothalamus

In mammals, the hypothalamic nuclei, located in the ventral diencephalon, play a pivotal role in the regulation of a number of vital physiological functions via direct synaptic stimulation of a wide range of targets or the secretion of various neuropeptides (Machluf et al., 2011). Homologs of diverse hypothalamic cell types secreting oxytocin, gonadotropin-releasing hormone, neuropeptide Y, and hypocretin have been identified in teleosts (Machluf et al., 2011). Hence it is likely that the zebrafish and terrestrial vertebrates have similar hypothalamic functions such as regulation of sleep, blood pressure, temperature, thirst and satiety, stress, reproduction, and social behavior. Mitral cells send direct projections to the hypothalamic area in fish but the exact localization of mitral cell terminals in hypothalamic nuclei and the functional significance of these projections remain unknown. In rodents, a ventral glomerulus projects to vasopressin or oxytocin secreting hypothalamic neurons (Hatton and Yang, 1989; Smithson et al., 1992; Bader et al., 2012). Vasopressin and oxytocin are known to modulate social behaviors in rodents as well as in fish (Godwin and Thompson, 2012). Olfactory cues are very important in signaling the presence of food or sexual partners in fish. The monosynaptic bulbo-hypothalamic projection in fish is therefore probably involved in the modulation of feeding and reproductive behaviors.

## CONCLUSION

Olfactory computations performed by the upstream olfactory brain areas in relation to behavior are well-documented in teleosts. Despite minor anatomical differences, the general principles and computations performed by the fish olfactory system are highly similar to what is described in terrestrial vertebrates. Odors are detected in a combinatorial manner by receptors expressed in olfactory sensory neurons. Olfactory sensory neurons expressing the same receptor are dispersed in the olfactory epithelium and project to one spatially confined glomerulus. Hence, the shuffled peripheral epithelial activation is reorganized into odor specific glomerular maps in the olfactory bulb. Odor-evoked activity patterns among mitral cell ensembles become less correlated with time, potentially helping discrimination of similar odorants. Ethologically relevant classes of odors tend to activate specific bulbar domains, resulting in a coarse topographic organization of different odor categories. Odor responses in the telencephalon do not seem to be topologically organized and the precise circuit mechanisms underlying the transformations of olfactory information in telencephalic targets still remain to be discovered. Odor responses in diencephalic areas such as the hypothalamus and habenula are currently not documented. Zebrafish lines that express calcium indicators in these areas are already available, which should allow the function of these areas to be revealed in the near future.

Currently, the neural pathways connecting the olfactory system to brain regions involved in the execution of different behaviors are not known in zebrafish. However, new techniques are rapidly being adopted which allow the tracing of functional connectivity in the olfacto-motor pathway. For example, the green fluorescent protein reconstitution across synaptic partners (GRASP) method, where non-fluorescent green fluorescent protein fragments expressed in two different neurons assemble to form the fluorophore at the synapse, is mainly used in invertebrates but is being adapted for vertebrates (Yamagata and Sanes, 2012). Additionally, links must be established between neural activity in olfactory bulb targets and relevant behavioral outputs. In this regard, the possibility of activating and deactivating genetically targeted neural populations offers new perspectives to understand how circuits elicit or modulate behavior. For example, the precise and non-invasive optogenetic stimulation of olfactory sensory neuron subsets already enables specific swimming patterns to be elicited in freely behaving zebrafish larvae (Zhu et al., 2009). In summary, thanks to its innate properties and a timely convergence of new techniques, the zebrafish olfactory system is an increasingly attractive model to understand the function of neural circuits involved in olfactory processing.

## Conflict of Interest Statement

The authors declare that the research was conducted in the absence of any commercial or financial relationships that could be construed as a potential conflict of interest.
